# Ross Procedure in the era of Handmade-Valved Conduits for Right Ventricular Outflow Tract Reconstruction in Children: Short-Term Surgical Outcomes

**DOI:** 10.3389/fcvm.2022.924253

**Published:** 2022-06-13

**Authors:** Wei Dong, Dian Chen, Qi Jiang, Renjie Hu, Lisheng Qiu, Hongbin Zhu, Wen Zhang, Haibo Zhang

**Affiliations:** Department of Cardiothoracic Surgery, Shanghai Children's Medical Center, Shanghai Jiaotong University School of Medicine, Shanghai, China

**Keywords:** Ross procedure, right ventricular outflow tract (RVOT), valved conduit, expanded polytetrafluoroethylene (ePTFE), surgical outcomes, children

## Abstract

**Objective:**

Ross procedure is considered as the “gold standard” for aortic valve replacement, but the conduits used for right ventricular outflow tract (RVOT) reconstruction, such as homografts and bovine jugular vein (BJV) conduits, are of limited availability in China. Handmade expanded polytetrafluoroethylene-valved conduits (HVCs) have been used recently as the alternative for RVOT reconstruction, but their specific experience in Ross procedure is limited in the literature.

**Methods:**

This was a retrospective review of 27 children who underwent Ross procedure in our center from January 2018 to January 2022.

**Results:**

Mean age at surgery was 8.0 ± 3.8 years. During the study period, BJV conduits were used for RVOT reconstruction in 6 patients (22%), and HVCs were used in 21 patients (78%). Median conduit size was 20 mm (range, 16–24 mm), and mean conduit Z-score was +0.8 ± 0.9. Median time for cardiopulmonary bypass was 158 min (range, 109–275 min), and mean time for aortic crossclamping was 110 ± 21 min. There was no early mortality. During a median follow-up time of 1.4 years (range, 0.1–3.7 years), 3 patients (11%) with BJV conduits had peak conduit velocity of > 3.5 m/s; 3 patients (11%) with HVCs developed moderate conduit insufficiency; no patients had more than moderate conduit insufficiency. Three patients with BJV conduits had 5 reinterventions, and all received conduit replacement with HVCs.

**Conclusion:**

HVC is an appealing alternative to BJV conduit for RVOT construction for children undergoing Ross procedure, with favorable short-term outcomes.

## Introduction

The Ross procedure is considered as the most attractive option for patients requiring aortic valve replacement, with excellent long-term survival (1). Although pulmonary autograft reoperations secondary to aortic valve insufficiency or aortic root dilation are sometimes needed, most reinterventions after Ross procedure are in the right ventricular outflow tract (RVOT) (2). A durable RVOT conduit is of great importance to the success of Ross procedure.

Homografts and bovine jugular vein (BJV) conduits have been widely used as RVOT conduits in western countries with favorable outcomes (3), but they are of limited availability in countries such as China. Handmade expanded polytetrafluoroethylene (ePTFE) valved conduits (HVCs) have been developed recently as an appealing alternative to homografts for RVOT reconstruction (4, 5), but the specific experience in Ross procedure in pediatric population is limited in the literature (6). Our institution has taken the approach of constructing HVC for RVOT reconstruction for Ross procedure since 2018. We now summarize our initial experience and evaluate the short-term surgical outcomes.

## Materials and Methods

### Patients

Between January 2018 and January 2022, 27 patients underwent Ross procedure at Shanghai Children's Medical Center and were included in this retrospective study. During this period, BJV conduits (BalMedic, China) were used for RVOT reconstruction in 6 patients (22%), all before March 2019; HVCs were used in 21 patients (78%). The hospital ethics committee approved this study and waived the need for individual consent (SCMCIRB-K2022033-1, April 6, 2022).

### Handmade-Valved Conduits

**Figure 1:**
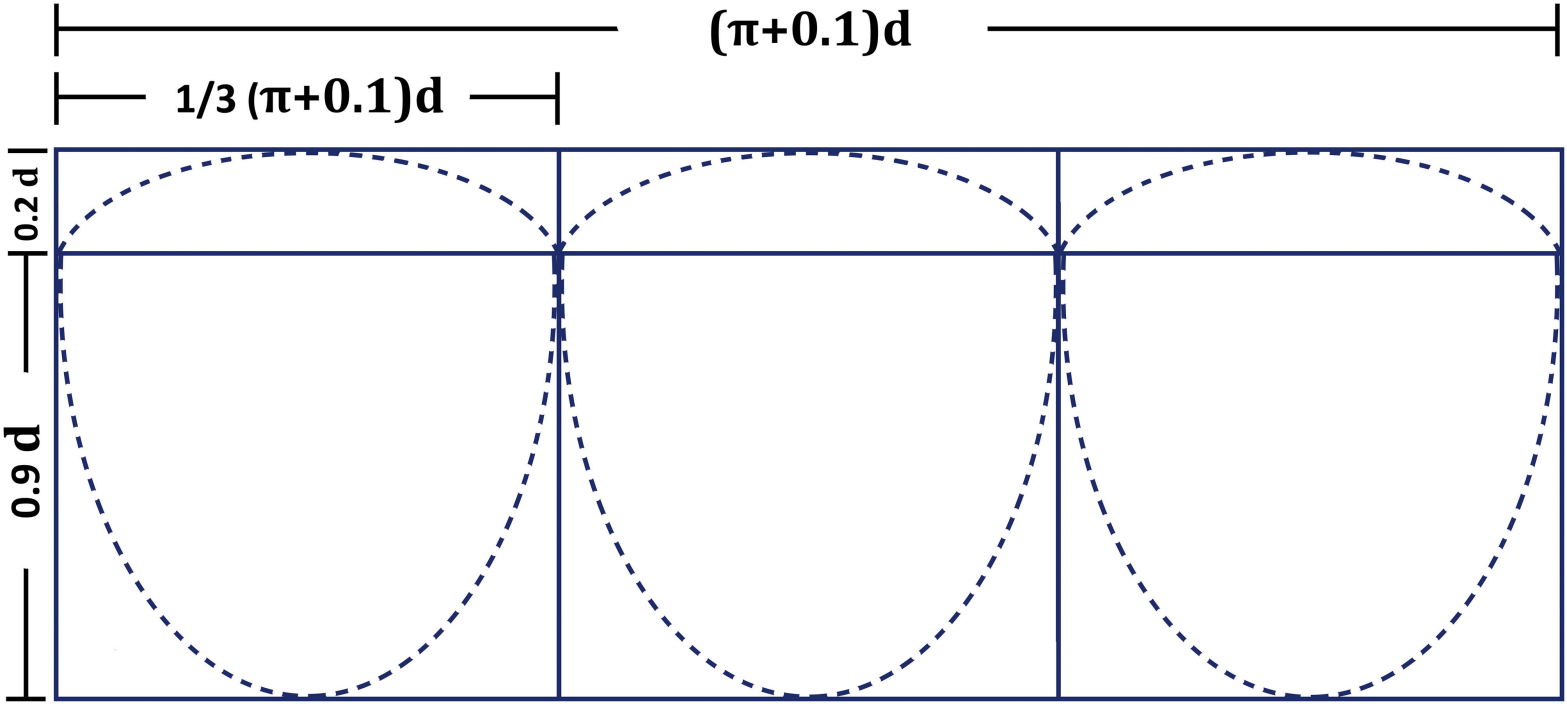
Measurements for the tricuspid handmade-valved conduit constructed with 0.1-mm-thick expanded polytetrafluoroethylene membrane. d, conduit diameter (mm).

The HVC was constructed in the operation room (by WD or HZhu) prior to skin incision (Supplementary Video). The technique was similar as previously described by Coyan and colleagues (7) with some modifications. Briefly, the conduit diameter was chosen based on age and weight of the patient. Due to the limited availability of different sizes of the conduits in our institution, different vascular grafts were used as conduits. Impra ePTFE vascular graft (BD, NJ) was used for HVC diameter of 16 and 19 mm. GORE-TEX vascular graft (W. L. Gore & Associates, Inc, AZ) was used for HVC diameter of 20 and 22 mm. Woven double velour vascular graft (Maquet, France) was used for HVC diameter of 24 mm and 26 mm. A 0.1-mm-thick ePTFE membrane (W. L. Gore & Associates, Inc, AZ) was trimmed to construct the tricuspid valve leaflets with the same shape (Figure 1), and were sewn on the surface of the inverted conduit with 6-0 prolene suture in a running fashion. Interrupted reinforcement sutures were placed on the commissures. The valved conduit was then inverted again and was tested for competency.

### Surgical Technique

The Ross procedure was performed by HZha or HZhu under cardiopulmonary bypass with aortobicaval cannulation. After inspection of the aortic valve, the decision was made to perform Ross procedure. The main pulmonary artery was transected at the bifurcation and the pulmonary autograft was harvested. The pulmonary autograft was then implanted to the aortic root with coronary transfer. The retained native aortic sinus tissue was used to cover the autograft as reinforcement. The HVC was then implanted to establish the right ventricle-to-pulmonary artery continuity.

### Follow-Up

Data were collected retrospectively from hospital records and outpatient clinics. Follow-ups were scheduled 1 month, 3 months, 6 months after discharge, then every 6 months for the next 18 months, and then every year. Echocardiograms were performed at each time points to evaluate the conduit performance. Conduit insufficiency was graded as none/trivial (0), mild (1), mild-moderate (1.5), moderate (2), moderate-severe (2.5), and severe (3) according to the features of the jet flow. Conduit dysfunction was defined as a peak conduit velocity 3.5 m/s or greater, or a > moderate conduit insufficiency, at the first occurrence (8).

### Statistical Analysis

Data were analyzed using SPSS software version 22.0 (IBM-SPSS Inc, Armonk, NY). Continuous variables were summarized using mean ± standard deviation or median (range) for skewness variables. Categorical variables were summarized as frequency and percentage. Comparison between continuous variables was performed by student's *t*-test, or by Mann–Whitney *U* test for skewness variables. Categorical variables were compared by the Fisher's exact test. The Cox proportional hazard analysis was used to determine the risk factor for conduit dysfunction. The *p* values of < 0.05 were considered statistically significant.

## Results

### Patient Characteristics

Mean age at surgery was 8.0 ± 3.8 years. Among them, the youngest patient was 11 months old, and 10 patients (37%) were more than 10 years old. Mean weight at surgery was 27.6 ± 11.8 kg, with eight patients (30%) weighing <20 kg. Eight patients (30%) had previous interventions on the aortic valve: 5 (63%) patients had percutaneous balloon aortic valvotomy and 3 (27%) patients had surgical aortic valvotomy. More than half of the patients (56%) had mixed aortic valve disease of stenosis and insufficiency. The detailed demographic characteristics were listed as Table 1. There were no significant differences in baseline characteristics between patients with BJV conduits and HVCs.

**Table 1:** Demographic characteristics of the entire cohort.

		**Overall**	**BJV conduit**	**HVC**	***P* value**
*N*		27	6 (22%)	21 (78%)	
Gender (female)		11 (41%)	3 (50%)	8 (38%)	0.662
Age (y)		8.0 ± 3.8	7.9 ± 2.8	8.0 ± 4.2	0.933
Weight (kg)		27.6 ± 11.8	28.3 ± 11.9	27.4 ± 12.0	0.868
Body surface area (m^2^)		1.0 ± 0.3	1.0 ± 0.3	1.0 ± 0.3	0.979
Aortic valve disease type	Stenosis	5 (18%)	1 (17%)	4 (19%)	0.802
	Insufficiency	7 (26%)	1 (17%)	6 (29%)	
	Mixed lesion	15 (56%)	4 (66%)	11 (52%)	
Etiology	Congenital	25 (93%)	6 (100%)	19 (90%)	1.000
	Endocarditis	2 (7%)	0	2 (10%)	
Aortic valve morphology	Tricuspid	15 (55%)	2 (33%)	13 (62%)	0.326
	Bicuspid	11 (41%)	4 (67%)	7 (33%)	
	Quadricuspid	1 (4%)	0	1 (5%)	
Previous intervention		8 (30%)	3 (50%)	5 (24%)	0.319
Conduit size (mm)		20 (16~24)	20 (18~20)	20 (16~24)	0.204
Conduit Z-score		+0.8 ± 0.9	+0.5 ± 0.9	+1.0 ± 0.8	0.215
Cardiopulmonary bypass time (min)		158 (109~275)	159 (123~258)	153 (109~275)	0.748
Aortic crossclamping time (min)		110 ± 21	125 ± 21	106 ± 19	0.048

*BJV, bovine jugular vein; HVC, handmade valved conduit*.

### Conduit Size

**Figure 2:**
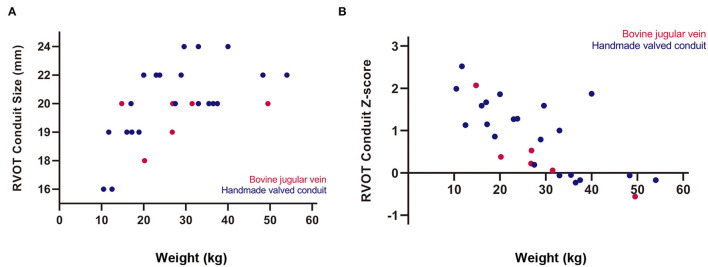
Relationships of body weight at surgery with **(A)** right ventricular outflow tract (RVOT) conduit size and **(B)** conduit *Z*-score.

Median conduit size was 20 mm (range, 16–24 mm), and mean conduit *Z*-score was +0.8 ± 0.9. Although there were no significant differences between the two groups in terms of conduit size and conduit *Z*-score; conduit size of 16, 22, and 24 mm was only selected in HVC (Table 2). Oversized conduit (*Z*-score >2) was implanted in 2 patients (7%), with the weight at the surgery of 12 and 15 kg, respectively. There was a downward trend of conduit *Z*-score with increasing weight at surgery (Figure 2).

**Table 2:** The selection of conduit size and conduit type in the entire cohort.

**Conduit size (mm)**	**Overall**	**BJV conduit**	**HVC**
16	2 (7%)	0	2 (9%)
18	1 (4%)	1 (17%)	0
19	5 (19%)	1 (17%)	4 (19%)
20	10 (37%)	4 (66%)	6 (29%)
22	6 (22%)	0	6 (29%)
24	3 (11%)	0	3 (14%)

*BJV, bovine jugular vein; HVC, handmade valved conduit*.

### Perioperative Outcomes

Three patients (11%) had concomitant aortic annular enlargement. Three patients (11%) had concomitant mitral valvuloplasty, and 1 patient (4%) had repair of double aortic arch. Median time for cardiopulmonary bypass was 158 min (range, 109–275 min), and mean time for aortic cross-clamping was 110 ± 21 min. Median time for mechanical ventilation and intensive care unit stay was 45 h (range, 10–163 h) and 4 days (range, 2–9 days), respectively. In-hospital complications occurred in 6 patients (22%): 2 patients had major bleedings, 1 patient with HVC had mediastinal infection, 1 patient with preoperative left ventricular dysfunction had delayed sternal closure, 1 patient had pericardial effusion requiring pericardial drainage, and 1 patient had postoperative moderate tricuspid regurgitation with moderate conduit insufficiency. There was no hospital mortality. After discharge, oral aspirin was administered in all patients. Warfarin was administered in 19 patients (90%) with HVCs for 6 months with the target of international normalized ratio (INR) of 1.5–2.0.

### Conduit Function

**Figure 3:**
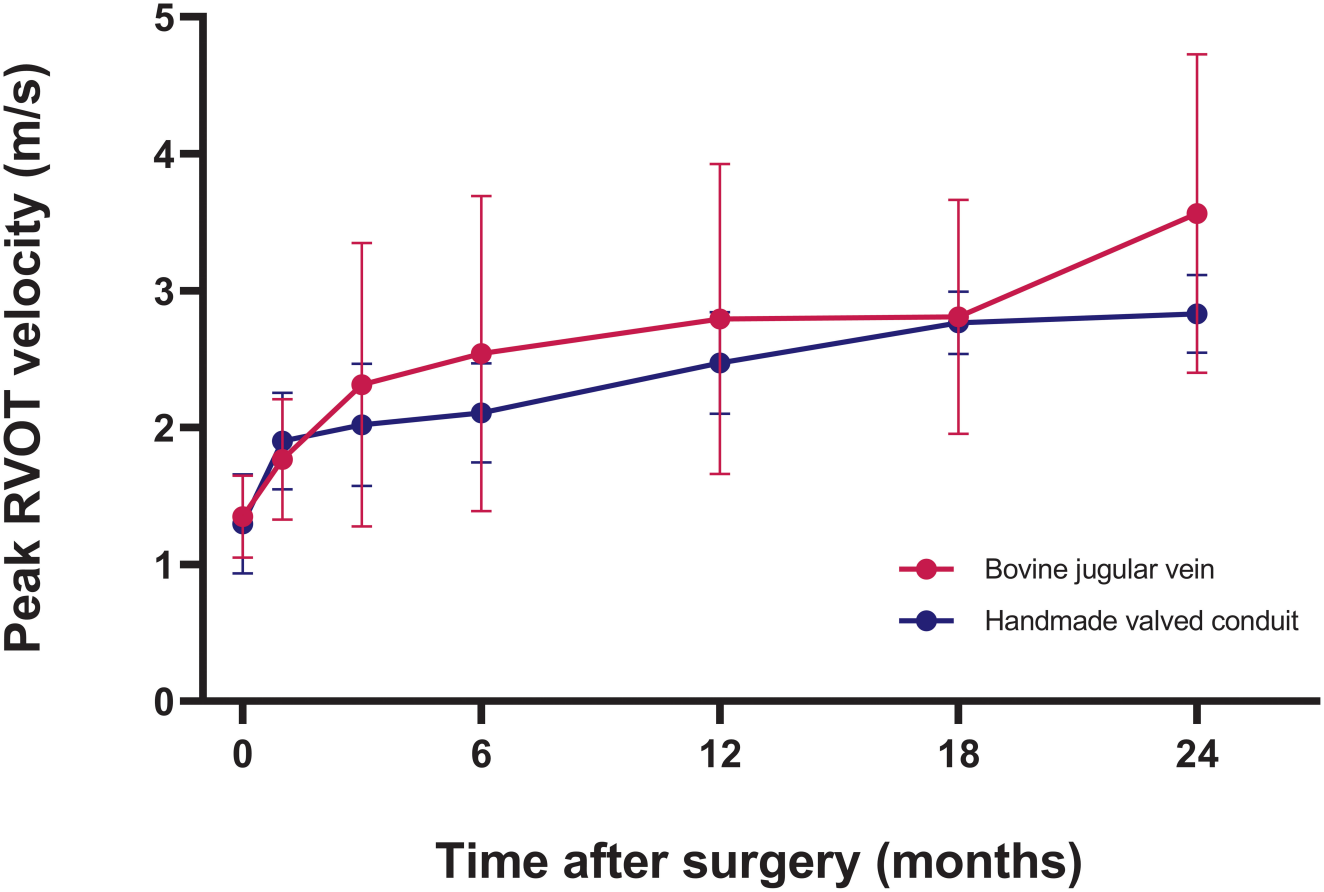
Graphic showing postoperative trend of peak right ventricular outflow tract (RVOT) velocity in two types of conduits.

**Figure 4:**
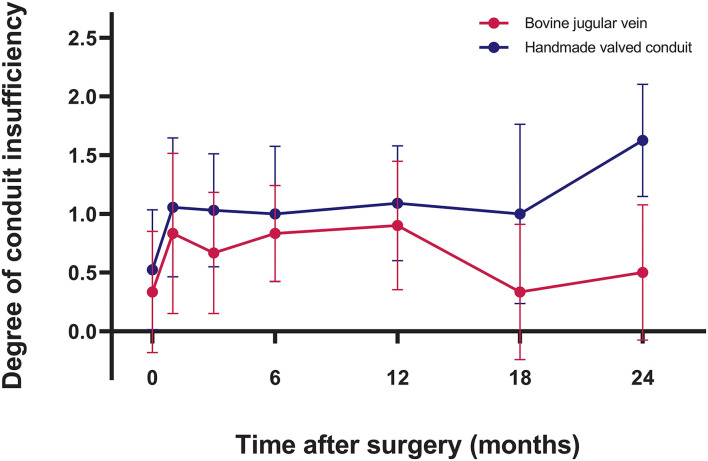
Graphic showing postoperative trend of degree of conduit insufficiency in two types of conduits.

Intraoperative transesophageal echocardiography showed none/trivial conduit insufficiency in 14 patients (52%), mild conduit insufficiency in 13 patients (48%), with no conduit stenosis. The median follow-up time was 1.4 years (range, 0.1–3.7 years). No patients were lost to follow-up. The mean peak conduit velocity at 1 year after surgery was 2.8 ± 1.1 m/s in BJV conduits, and 2.5 ± 0.4 m/s in HVCs (*p* = 0.561) (Figure 3). Three patients (11%) with BJV conduits had peak conduit velocity of > 3.5 m/s; all occurred at the distal anastomosis of the conduit. The mean degree of conduit insufficiency at 1 year after surgery was 0.9 ± 0.5 in BJV conduits, and 1.1 ± 0.5 in HVCs (*p* = 0.497) (Figure 4). Three patients (11%) with HVCs developed moderate conduit insufficiency; no patients had more than moderate conduit insufficiency. No risk factor for conduit dysfunction was identified by the Cox proportional hazard analysis (Table 3).

**Table 3:** Univariable risk factors for conduit dysfunction after Ross procedure.

**Univariable risk factor**	**Hazard ratio (95% confidence interval)**	***P*** **value**
Gender	1.231 (0.11~13.652)	0.865
Age	1.287 (0.829~1.997)	0.260
Weight	1.071 (0.957~1.199)	0.232
Body surface area	17.508 (0.098~3136.864)	0.280
Aortic valve disease type	2.415 (0.284~20.521)	0.419
Aortic valve morphology	0.491 (0.048~5.080)	0.551
Etiology	0.030 (0.000~80925.479)	0.642
Previous intervention	0.998 (0.090~11.013)	0.999
Conduit type	0.006 (0.000~218.911)	0.338
Conduit size	0.826 (0.393~1.739)	0.826
Conduit Z-score	0.055 (0.001~3.917)	0.183
Cardiopulmonary bypass time	1.021 (0.996~1.047)	0.105
Aortic crossclamping time	1.050 (0.991~1.112)	0.095

### Conduit Reintervention

Three patients with BJV conduits had 5 reinterventions on the conduit during the follow-up period. No patients with HVCs required reintervention. The first patient was a 12-year-old, 32 kg male with initial BJV conduit of 20 mm (*Z*-score: +0.06). He developed severe conduit stenosis (peak conduit velocity: 4.3 m/s) requiring percutaneous balloon dilation of the distal conduit 6 months after initial surgery. After surgery the stenosis progressed and at 2.8 years after initial surgery, he received conduit replacement with HVC of 26 mm (*Z*-score: +1.16). The pulmonary artery bifurcation was enlarged with pericardial patch. This patient died of cerebral hemorrhage unrelated to the conduit. The second patient was a 10-year-old, 49 kg male with initial BJV conduit of 20 mm (*Z*-score: −0.56). He had percutaneous balloon dilation of distal conduit at 8 months and subsequent conduit replacement with HVC of 20 mm (*Z*-score: −0.91) at 1.5 years after initial surgery, respectively. The third patient was 6-year-old, 20 kg female with initial BJV conduit of 18 mm (*Z*-score: +0.38). She had conduit replacement with HVC of 22 mm (*Z*-score: +0.78), enlargement of the pulmonary artery bifurcation with pericardial patch, aortic valvuloplasty at the right-noncoronary commissure, and replacement of the ascending aorta with ePTFE conduit at 2.8 years after surgery. The explants of the above-three BJV conduits showed conduit calcification as well as neointimal proliferation at the distal anastomosis.

## Discussion

The Ross procedure is considered by many as the “gold standard” for aortic valve replacement with excellent hemodynamics, durability, and free from anticoagulation therapy (1). However, reintervention after the Ross procedure is still a concern, with reoperation due to pulmonary autograft and RVOT conduit failure occurred at the rates of 0.37–2.81%/year and 0.34–4.76%/year, respectively (9). Implanting an RVOT conduit with a durable conduit function will reduce the burden for reinterventions after the Ross procedure.

Younger age at surgery, lower weight at surgery and smaller conduit size at implantation have already been identified as the risk factors for RVOT reoperation due to somatic growth (9, 10). Homografts and BJV conduits are the two most frequently used conduits for RVOT reconstruction (3, 10). Homografts have been considered as the gold standard for RVOT reconstruction, but with limited availability especially in smaller sizes. BJV conduits on the other hand are available “off-the-shelf,” and have been widely used as the alternative to homografts (10). In a recent multicenter study comparing homografts and BJV conduits in patients <20 years of age, homografts outperformed BJV conduits in patients with a conduit size of larger than 15 mm (8). In a retrospective study of four different RVOT conduits used in a single institution over 30 years, homografts had greater durability than BJV conduits, especially in patients weighing 5 to 20 kg (10). However, the performance of homografts and BJV conduits seems to be comparable in patients with Ross procedure. Patel and colleagues (11) reported similar postoperative peak gradient of the conduit, degree of conduit insufficiency, and freedom from pulmonary valve replacement between patients with homografts and BJV conduits.

However, homografts and BJV conduits are of limited availability in some Asian countries. Miyazaki and colleagues from Japan first developed a novel handmade tricuspid-valved conduit with a fan-shaped configuration using a 0.1-mm-thick ePTFE membrane that could be constructed in the operation room. Three bulging sinuses were added to improve the closing motion of the valves on the inner surface of the sinuses (12). Centers outside Japan generally use HVC without the bulging sinus, because the construction of a bulging sinus requires special device, and its existence may be nonessential in the pulmonary position (5, 13). Other groups made various modifications on the valve configuration (7, 14, 15), or developed special templates for tailoring the valve (13). Since 2018, our institution began to use HVC as an RVOT conduit for Ross procedure, with technical modifications. Our simplified approach increases the effective height of the valve leaflet to decrease the risk of postoperative regurgitation, and its configuration can be easily calculated. The whole construction process generally takes about 20–25 min.

The performance of HVCs has been demonstrated with promising durability. In a recent multicenter study from Japan involving 1,776 patients undergoing RVOT reconstruction with HVCs, freedom from reintervention was 86.7% at 5 years and 61.5% at 10 years (5). Relative conduit stenosis due to somatic growth was the most common reason for conduit explantation (5). Notably, in patients with large conduit size at implantation, freedom from reintervention was 98.9% at 5 years and 88.4% at 10 years, suggesting its long-term durability (16). Intervention for stenosis at the distal anastomosis was uncommon compared to BJV conduits (5). Similarly, HVC regurgitation is more likely to occur in smaller conduit, but within acceptable values (5). In our cohort, 3 patients had moderate conduit regurgitation during the follow-up period, all occurred during the early study period, probably related to the learning curve of HVC construction. HVC dysfunction is characterized by calcification and neointimal proliferation (14, 17). Yamamoto et al. (17) examined the explanted HVCs due to conduit failure. Leaflet calcification tended to occur in the middle and upper third of the leaflet, leading to stiffening and distortion of the leaflet, with neointimal proliferation on the leaflet. Most leaflets of the dysfunctional conduit were fixed either in an open position or a semi-closed position (5, 17). Of note, none of these conduits were implanted for the Ross procedure. As the performance of RVOT conduits in patients with Ross procedure are generally thought to be superior to other diseases such as truncus arteriosus (10), it can be inferred that HVC is the optimal alternative to homograft or BJV conduit for Ross procedure. Since March 2019, we no longer use BJV conduits for the Ross procedure.

There is continuing debate over the selection of primary vs. secondary Ross procedure for patients with aortic valve disease. The survival and freedom from reintervention are superior when Ross procedure is performed in older children compared to neonates or infants (18). Some suggested that the approach of postponing Ross procedure could achieve better outcomes than performing primary Ross procedure (19). Our center follows the same policy, which may also reduce the need for RVOT conduit replacement due to somatic growth. The size of the RVOT conduit for Ross procedure is generally oversized (9–11), but larger conduit at implantation may not always be feasible given the limited volume of the thoracic cavity. There were reported cases where the conduits were compressed by the sternum (20). The youngest patient in our cohort was an 11-month-old, 10.5 kg male with HVC diameter of 16 mm (*Z*-score: +1.99). It is also the smallest ePTFE conduit available in our institution. In our experience, for patients weighing <15 kg, the conduit *Z*-score would be around +2 to +3. Conduit *Z*-score around +1 to +2 would be suitable for patients weighing between 15 and 30 kg, while normal conduit size for those weighing more than 30 kg.

Anticoagulation is required after HVC implantation in Ross procedure, which is an obvious drawback for a procedure that is originally designed to avoid anticoagulation. Anticoagulation strategy after HVC implantation has not reached a consensus, and is mainly based on the institutional preference. In Japan, anticoagulation therapy generally consisted of oral aspirin for 6 months after HVC implantation (5). Most patients with conduit size of 14 mm or greater were free of any anticoagulants after 6 months. Warfarin was only prescribed additionally to patients with conduit size of 8 mm, with INR controlled between 1.5 and 2.0. In a study from Korea, warfarin was prescribed after the restart of oral intake, and was continued for 3 months. Aspirin was administered indefinitely thereafter (14). One group from China reported their experience of using warfarin during the first 6 months with the target INR between 1.5 and 2.0, and oral aspirin for 2 years (13). This policy was similar to ours, but oral aspirin was still continued after 2 years in our practice. In a prospective, multicenter study of a novel ePTFE-valved conduit, which did not include any coating on the conduit, aspirin was administered in all patients with minimum of 3 months after surgery, and temporary warfarin was only given to 24% of the patients (21). Symptomatic embolism event, valve thrombosis, and bleeding event associated with HVC were not reported from any of the above groups. The investigation of the optimal anticoagulation strategy for patients with HVC implantation is challenging given its low incidence of morbidity, and it requires a multicenter randomized-controlled trial with a large sample size.

### Limitations

This was a single-center retrospective study with limited sample size and short period of follow-up. Longer-term follow-up study is needed to verify the durability of our HVCs. Three different types of vascular grafts were used to construct HVCs of different sizes, which is a confounding factor when analyzing the conduit performance. Given the small sample size and the small number of events, no risk factors were identified.

## Conclusion

HVC is an appealing alternative to BJV conduit for RVOT construction for children undergoing Ross procedure, with favorable short-term outcomes.

## Data Availability Statement

The datasets presented in this article are not readily available because Ethical restrictions. Requests to access the datasets should be directed to HZha, zhanghaibosh@126.com.

## Ethics Statement

The studies involving human participants were reviewed and approved by Shanghai Children's Medical Center, Shanghai Jiaotong University School of Medicine. Written informed consent from the participants' legal guardian/next of kin was not required to participate in this study in accordance with the national legislation and the institutional requirements. Written informed consent was not obtained from the minor(s)' legal guardian/next of kin for the publication of any potentially identifiable images or data included in this article.

## Author Contributions

WD and DC were responsible for conceptualization, data curation, and writing the manuscript. QJ was responsible for data curation and statistical analysis. RH was responsible for patients' follow-up. LQ and HZhu were responsible for reviewing and editing. WZ and HZha were responsible for conceptualization, reviewing, editing, and supervision. All authors contributed to the article and approved the submitted version.

## Funding

This study was supported by the Natural Science Foundation of China (82070322), Shanghai Municipal Science and Technology Commission Research Project (19411950200), and Shenkang Cutting-Edge Research Project (SHDC12018128).

## Conflict of Interest

The authors declare that the research was conducted in the absence of any commercial or financial relationships that could be construed as a potential conflict of interest.

## Publisher's Note

All claims expressed in this article are solely those of the authors and do not necessarily represent those of their affiliated organizations, or those of the publisher, the editors and the reviewers. Any product that may be evaluated in this article, or claim that may be made by its manufacturer, is not guaranteed or endorsed by the publisher.

## References

[1] Wiggins LM Kumar SR Starnes VA. The Ross procedure in children: The gold standard? Semin Thorac Cardiovasc Surg Pediatr Card Surg Annu. (2021) 24:62–6. 10.1053/j.pcsu.2021.03.002 34116784

[2] Martin E Laurin C Jacques F Houde C Cote JM Chetaille P. More than 25 years of experience with the Ross procedure in children: a single-center experience. Ann Thorac Surg. (2020) 110:638–44. 10.1016/j.athoracsur.2019.10.093 31881194

[3] Herrmann JL Brown JW. Seven decades of valved right ventricular outflow tract reconstruction: The most common heart procedure in children. J Thorac Cardiovasc Surg. (2020) 160:1284–8. 10.1016/j.jtcvs.2020.04.137 32690409

[4] Yamagishi M. Right ventricular outflow reconstruction using a polytetrafluoroethylene conduit with bulging sinuses and tricuspid fan-shaped polytetrafluoroethylene valve. Oper Tech Thorac Cardiovasc Surg. (2016) 21:211–29. 10.1053/j.optechstcvs.2017.05.00217662769

[5] Hongu H Yamagishi M Maeda Y Itatani K Fujita S Nakatsuji H. Expanded polytetrafluoroethylene conduits with bulging sinuses and a fan-shaped valve in right ventricular outflow tract reconstruction. Semin Thorac Cardiovasc Surg. (2021) 10.1053/j.semtcvs.2021.02.026. [Epub ahead of print]. 33691193

[6] Sharifulin R Bogachev-Prokophiev A Demin I Zheleznev S Pivkin A Afanasyev A. Right ventricular outflow tract reconstruction using a polytetrafluoroethylene conduit in Ross patients. Eur J Cardiothorac Surg. (2018) 54:427–33. 10.1093/ejcts/ezy128 29659782

[7] Coyan G Da Fonseca Da Silva L Da Silva J Viegas M Morell V Castro-Medina M. Polytetrafluoroethylene tricuspid valved conduit for right ventricular outflow tract reconstruction. Ann Thorac Surg. (2021) 111:e455–e8. 10.1016/j.athoracsur.2020.11.090 33631155

[8] Marathe S Hussein N Wallace F Bell D Yong M Betts K. Comparison of homografts and bovine jugular vein conduits in the pulmonary position in patients <20 years of age. J Thorac Cardiovasc Surg. (2021) 21:S0022–5223 10.1016/j.jtcvs.2021.11.087 35058063

[9] Moroi MK Bacha EA Kalfa DM. The Ross procedure in children: a systematic review. Ann Cardiothorac Surg. (2021) 10:420–32. 10.21037/acs-2020-rp-23 34422554 PMC8339620

[10] Willetts R Stickley J Drury N Mehta C Stumper O Khan N. Four right ventricle to pulmonary artery conduit types. J Thorac Cardiovasc Surg. (2021) 162:1324–33.e3. 10.1016/j.jtcvs.2020.12.144 33640135

[11] Patel PM Herrmann JL Rodefeld MD Turrentine MW Brown JW. Bovine jugular vein conduit versus pulmonary homograft in the Ross operation. Cardiol Young. (2020) 30:323–7. 10.1017/S1047951119003007 31847922

[12] Miyazaki T Yamagishi M Maeda Y Yamamoto Y Taniguchi S Sasaki Y. Expanded polytetrafluoroethylene conduits and patches with bulging sinuses and fan-shaped valves in right ventricular outflow tract reconstruction: multicenter study in Japan. J Thorac Cardiovasc Surg. (2011) 142:1122–9. 10.1016/j.jtcvs.2011.08.018 21908008

[13] Shi Q Shan Y Chen G Mi Y Zhong H Jia B. Midterm outcomes for polytetrafluoroethylene valved conduits. Ann Thorac Surg. (2021) 1:S0003–4975. 10.1016/j.athoracsur.2021.09.051 34717907

[14] Choi KH Sung SC Kim H Lee HD Kim G Ko H. Simplified tricuspid polytetrafluoroethylene valved conduit: Midterm results of multicenter study. Ann Thorac Surg. (2019) 108:1228–33. 10.1016/j.athoracsur.2019.04.018 31102636

[15] Ando M Takahashi Y. Ten-year experience with handmade trileaflet polytetrafluoroethylene valved conduit used for pulmonary reconstruction. J Thorac Cardiovasc Surg. (2009) 137:124–31. 10.1016/j.jtcvs.2008.08.060 19154914

[16] Fujita S Yamagishi M Miyazaki T Maeda Y Itatani K Yamamoto Y. Long-term results of large-calibre expanded polytetrafluoroethylene-valved conduits with bulging sinuses. Eur J Cardiothorac Surg. (2020) 58:1274–80. 10.1093/ejcts/ezaa240 32984875

[17] Yamamoto Y Yamagishi M Maeda Y Asada S Hongu H Fujita S. Histopathologic analysis of explanted polytetrafluoroethylene-valved pulmonary conduits. Semin Thorac Cardiovasc Surg. (2020) 32:990–9. 10.1053/j.semtcvs.2019.10.001 31606427

[18] Buratto E Konstantinov IE. Aortic valve surgery in children. J Thorac Cardiovasc Surg. (2020). 10.1016/j.jtcvs.2020.06.145. [Epub ahead of print]. 32891449

[19] Donald JS Wallace FRO Naimo PS Fricke TA Brink J Brizard CP. Ross operation in children: 23-year experience from a single institution. Ann Thorac Surg. (2020) 109:1251–9. 10.1016/j.athoracsur.2019.10.070 31863757

[20] Miyazaki T Yamagishi M Maeda Y Taniguchi S Fujita S Hongu H. Long-term outcomes of expanded polytetrafluoroethylene conduits with bulging sinuses and a fan-shaped valve in right ventricular outflow tract reconstruction. J Thorac Cardiovasc Surg. (2018) 155:2567–76. 10.1016/j.jtcvs.2017.12.137 29510932

[21] Baird C Chávez M Backer C Galantowicz M Del Nido P. Preliminary results with a novel expanded polytetrafluoroethylene-based pulmonary valved conduit. Ann Thorac Surg. (2021) 1:S0003–4975. 10.1016/j.athoracsur.2021.10.033 34838744

